# Driving Ability in Alzheimer Disease Spectrum: Neural Basis, Assessment, and Potential Use of Optic Flow Event-Related Potentials

**DOI:** 10.3389/fneur.2018.00750

**Published:** 2018-09-07

**Authors:** Takao Yamasaki, Shozo Tobimatsu

**Affiliations:** ^1^Department of Clinical Neurophysiology, Neurological Institute, Graduate School of Medical Sciences, Kyushu University, Fukuoka, Japan; ^2^Department of Neurology, Minkodo Minohara Hospital, Fukuoka, Japan

**Keywords:** Alzheimer disease spectrum, radial optic flow perception, event-related potentials, driving ability, Alzheimer's disease, mild cognitive impairment

## Abstract

Driving requires multiple cognitive functions including visuospatial perception and recruits widespread brain networks. Recently, traffic accidents in dementia, particularly in Alzheimer disease spectrum (ADS), have increased and become an urgent social problem. Therefore, it is necessary to develop the objective and reliable biomarkers for driving ability in patients with ADS. Interestingly, even in the early stage of the disease, patients with ADS are characterized by the impairment of visuospatial function such as radial optic flow (OF) perception related to self-motion perception. For the last decade, we have studied the feasibility of event-related potentials (ERPs) in response to radial OF in ADS and proposed that OF-ERPs provided an additional information on the alteration of visuospatial perception in ADS (1, 2). Hence, we hypothesized that OF-ERPs can be a possible predictive biomarker of driving ability in ADS. In this review, the recent concept of neural substrates of driving in healthy humans are firstly outlined. Second, we mention the alterations of driving performance and its brain network in ADS. Third, the current status of assessment tools for driving ability is stated. Fourth, we describe ERP studies related to driving ability in ADS. Further, the neural basis of OF processing and OF-ERPs in healthy humans are mentioned. Finally, the application of OF-ERPs to ADS is described. The aim of this review was to introduce the potential use of OF-ERPs for assessment of driving ability in ADS.

## Introduction

Driving is a complicated skill that needs to integrate multiple cognitive, perceptual and motor abilities (3), and is supported by widely distributed brain network responsible for these complex processes (4–8). The driving ability can be disturbed by a decline in these brain networks due to normal aging and cognitive impairment such as dementia (3, 9–11). In recent years, the number of individuals with dementia is steadily increasing due to aging of the population (12). Under such circumstances, traffic accidents in individuals with dementia have increased and become an urgent social problem (11).

Among dementia, Alzheimer's disease (AD) is the most common (12). AD progresses on a spectrum with three stages, so-called, “AD spectrum (ADS)” (13); (1) preclinical AD (14), (2) mild cognitive impairment (MCI) due to AD (15), and (3) AD dementia (16). AD dementia is characterized by the impairment of short-term episodic memory, orientation, visuospatial function, language and executive function (12). The major neuropathological hallmarks of AD are deposition of β amyloid (senile plaques) and accumulation of neurofibrillary tangles, which cause a series of toxic events that result in synaptic dysfunction, neuronal loss and brain atrophy (12). Overall, multiple cognitive function associated with distributed brain network are impaired due to the AD pathology, resulting in the decline of driving ability in patients with AD.

There are various methods to assess driving ability, which include on-road test, driving simulation, and neuropsychological tests. However, recent systematic review and meta-analysis on these methods have demonstrated a lack of consistency of the findings among the studies though the several cognitive tests are considered to be the predictors of driving performance in AD patients (3, 17). So far, there have been no tests sufficient to determine driving safety, so it is necessary to establish a reliable method that can accurately evaluate driving ability in ADS. Interestingly, visuospatial dysfunction is often an early symptom even in the early stage of ADS (18, 19). Specifically, psychophysical studies demonstrated that AD patients exhibited selective elevation of motion coherence thresholds for radial optic flow (OF) motion which was related to self-motion perception (20), compared with those of coherent horizontal (HO) motion and static forms (19). In addition, the impaired OF perception was correlated with poor performance of the spatial navigation test (19). These findings suggest that the deficits of OF perception is responsible for the impairment of spatial navigation including the driving performance in AD patients. Some patients with MCI also exhibited selective impairment of coherent OF motion perception (18).

Event-rerated potentials (ERPs) are a pertinent tool to assess the visual function as well as dysfunction in humans because ERPs are non-invasive, objective, rapid, repeatable with the low cost. ERPs are also characterized by excellent temporal resolution (<1 ms) and can measure neural activity directly compared with functional magnetic resonance imaging (fMRI) (21, 22). Therefore, radial OF-ERPs may be a neural biomarker for decline of driving performance in ADS.

In this review, we first outline the neural basis of driving ability in healthy humans. Second, we describe the alterations of performance and associated brain function for driving in ADS. Third, we refer to current status of the assessment tests for driving and its problems. Fourth, ERP studies related to driving ability in ADS are stated. Further, we mention the neural basis of OF perception and findings of OF-related ERPs in healthy humans. Finally, we introduce the potential use of OF-ERPs for assessing driving ability of ADS. The aim of this review was to stress the feasibility of neurophysiological evaluation of OF perception that can be a neural biomarker for altered driving ability in ADS.

## Neural basis of driving ability in healthy individuals

**Figure 1:**
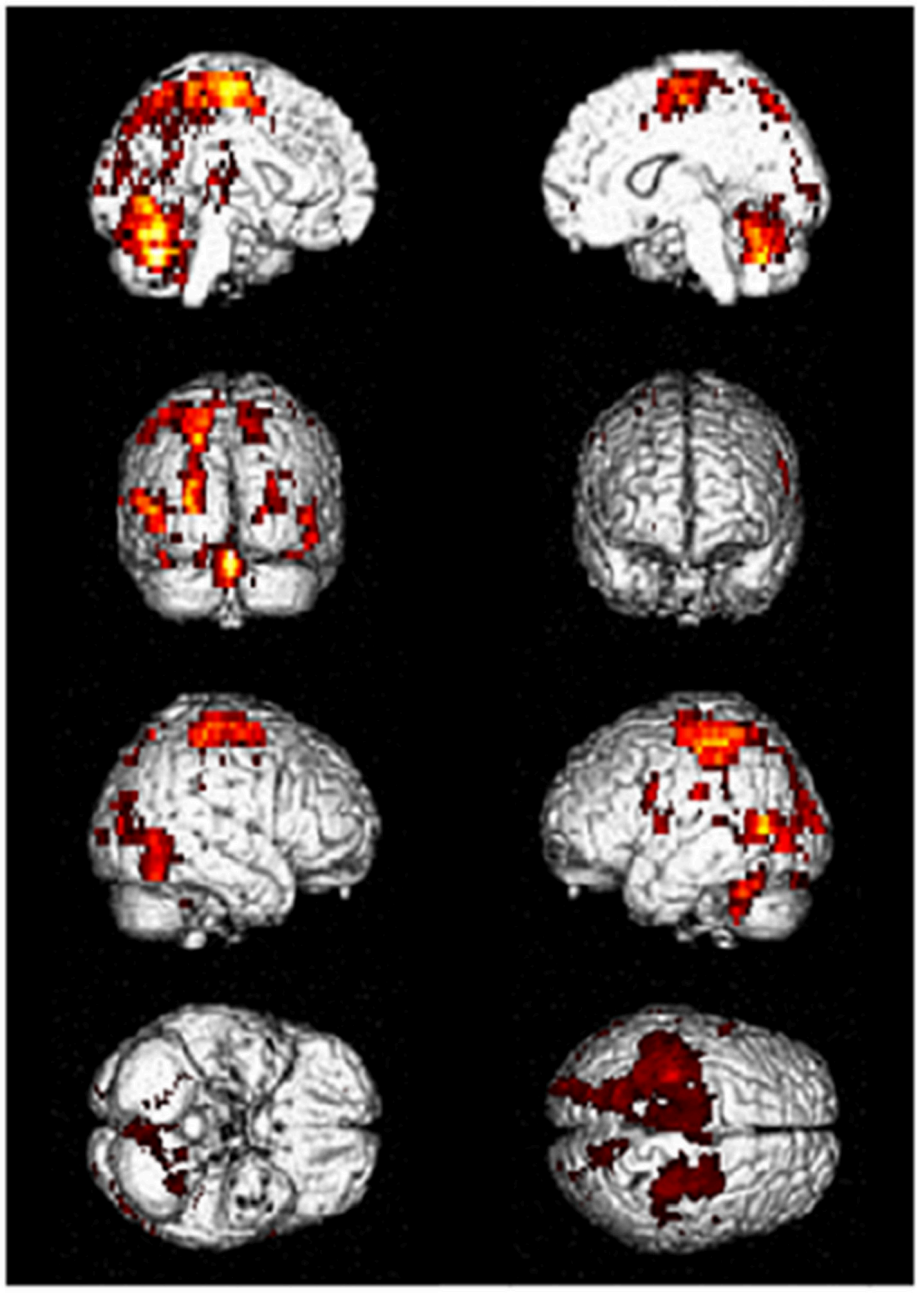
Activated brain regions during driving in fMRI. Distributed brain networks including occipital, parietal, frontal, motor, and cerebellar regions are mainly activated while driving only task. fMRI, functional magnetic resonance imaging. [Modified from (4), licensed under Creative Commons].

Driving requires the coordination of multiple cognitive functions and recruitment of associated multiple brain regions. Several fMRI studies on various driving tasks have demonstrated the activation of widespread brain network including occipital, parietal, frontal, motor and cerebellar regions and others to maintain safe driving (4–8). Figure 1 shows an example of activated brain regions while driving in a recent fMRI study (4). In their study, during driving only condition, the occipital activations were observed in the inferior, superior and middle occipital gyri and lingual gyrus. The activated areas of parietal lobe were superior and inferior parietal lobe, postcentral gyrus, and precuneus. The activations of frontal regions consisted of the inferior, middle and superior frontal gyri and precentral gyrus. The superior and middle temporal gyri were the activated areas of temporal regions. The activations of the cerebellum included the uvular, declive, and cerebellar tonsil. In addition, the limbic region such as cingulate gyrus, sub-lobar region including insula and lentiform nucleus were activated (4).

During driving, occipital and parietal regions plays a crucial role in visuospatial perception and attention to visual motion and fixed landmarks during vehicle movement. The frontal region is important for the executive function, working memory, processing thoughts, and decision-making. The motor and cerebellar regions engage in fine-control and action planning during movement execution (4–8). Furthermore, the recruitment of these brain regions is changeable but not uniform while driving. For instance, during distracted driving, brain activations shift the posterior regions to the frontal regions, particularly in the prefrontal areas (6). Taken together, because brain networks related to driving are broadly distributed, they may be susceptible to brain disorder such as ADS which shows extensive brain damage.

## Altered driving performance in ADS

Older drivers are at higher risk for traffic accidents such as crashes, injuries and deaths than other age groups (11). Further, individuals with AD dementia have an increased risk of traffic accidents compared to healthy older drivers (11). Severity of decline in driving performance was correlated with a degree of cognitive impairment in AD dementia (23). Individuals with MCI also had significantly more errors (collisions, center line crossings, road edge excursions, stop sign missed, speed limit exceedance) compared with healthy control drivers (10). MCI is classified into two types: amnestic MCI (aMCI) (with memory impairment) and non-aMCI (without memory impairment) (24). MCI is further classified into single-domain MCI (with impairment in single cognitive domain) and multiple-domain MCI (with impairment in multiple cognitive domain) (24). Patients with multiple-domain aMCI have the two or more impairments of memory, attention, viusospatial function, and executive function. Comparing multiple-domain aMCI with single-domain aMCI, the former demonstrates greater driving difficulty compared with the latter and healthy controls (10). Since all these cognitive functions are important for driving performance, multiple-domain aMCI may exhibit a greater driving difficulty than single-domain aMCI.

A single-photon emission computed tomography (SPECT) study has demonstrated that severity of impaired driving performance is significantly correlated with the changes of cerebral blood flow in the temporo-parietal regions in early stage of AD (25). A positron emission tomography (PET) study showed that the executive functioning was correlated with metabolism in the temporo-parietal regions, which was impaired in early stage of AD (26). Neuropsychological studies also reported a significant relationship between driving performance and visuospatial perceptual ability in AD (17). These findings indicate that the hypoperfusion or hypometabolism of temporo-parietal regions reflects the impairments of visuospatial perception and executive function, which result in impaired driving performance in early stage of AD. Moreover, with increased severity of driving impairment, the perfusion of frontal region was also reduced in addition to temporo-parietal regions in SPECT (25). The AD pathology is observed in the temporo-parietal regions in the early stage of the disease while that pathology spread into the frontal regions in the later stage (27). Therefore, the impairment of executive function involving the frontal regions can be more correlated with the driving impairment for the late stage of AD. Interestingly, a recent PET study have revealed that driving risk is strongly correlated with accumulation of amyloid even in the preclinical stage of AD (28). In another study using tau and amyloid PET, participants at Stage 2 [amyloid (+) and tau (+)] of preclinical AD (14) were more likely to receive a marginal/fail rating compared to participants at Stage 0 [amyloid (-) and tau (-)] or 1 [amyloid (+) and tau (-)] (11, 14). This finding suggests that individuals with preclinical AD (Stage 2) may already decline in driving skills.

Overall, the driving performance is gradually worsening along with the course of ADS from preclinical AD to AD dementia. These alterations of driving performance seem to be induced by the progression of AD pathology. In particular, the early pathological change in the posterior temporo-parietal regions associated with visuospatial function (OF perception) may be responsible for the impaired driving in the early stage of ADS.

## Assessment tools for driving ability in ADS

Various methods including on-road test, driving simulation and neuropsychological tests have been used for evaluating driving ability (3, 17, 29). The on-road test is the gold standard for assessing fitness to drive, but it requires much time for patients. There is also a need for someone who is proficient in the judgment. The driving simulation is similar to the on-road test, but it is expensive. Therefore, these two tests cannot be routinely performed at the medical clinics. For this reason, neuropsychological tests are commonly used. Neuropsychological tests can evaluate various aspects of brain function including attention, executive function and visuospatial abilities known to be impaired in patients with ADS. For example, the following tests are frequently used; the Mini-Mental State Examination (MMSE) for memory, attention and language skill, the Trail Making Test Part A and B (TMT-A and -B) for cognitive flexibility, Drawing test for visuo-constructive ability, and Maze test for visual orientation (29). However, these neuropsychological examinations, especially when doing multiple tests, require a long time to perform, so that patients often get tired. Characteristics with some pros and cons of these assessment tools are briefly summarized in Table 1.

**Table 1:** Assessment tools for driving ability in ADS.

**Assessment tools**	**Characteristics**	**Pros**	**Cons**
On-road test	- Gold standard - Evaluate driving abilities using actual vehicle by a trained expert	- Close to driving in the natural environment	- Expensive - Limited availability - Need a trained expert - Long time to perform - Cannot examine the driving ability under hazardous conditions
Driving simulators	- Mimic real-world driving using a front monitor, a handle, an accelerator, a brake pedal, etc. which resemble an actual vehicle	- Wide range of test conditions (e.g., night and day, different weather conditions, or road environments) - Especially, we can safely examine the driving performance under hazardous conditions	- Expensive - Limited availability
Neuropsychological tests	- Assess various cognitive functions indispensable for driving (e.g., attention, executive function and visuospatial abilities, etc)	- Widely available - Multiple options for standardized measures	- Long time to perform - Need a trained expert
ERPs	- Directly measure neural activity from scalp electrodes while watching OF stimuli in the case of OF-ERPs	- Widely available - Non-invasive - Inexpensive - Short time to perform - Easy to use	- Currently not standardization for driving assessment

*ERPs, event-related potentials; OF, optic flow*.

There have been many studies that investigate the usefulness of above mentioned tests as predictors of driving ability (3, 17, 29). However, a recent systematic review (17) demonstrated a lack of consistency in the findings, with some studies showing a relationship between cognitive test and driving performance for individuals with AD, whereas others did not. Further, this review suggested that deficits in a single cognitive ability were not a reliable predictor of driving performance. In contrast, a composite battery that assessed the multiple cognitive domains required to be an efficient driver was the best predictor of driving performance in individuals with AD (17). Another study compared the predictive value of the three types of assessment such as clinical interview, neuropsychological test battery (including multiple tests) and driving simulation (29). They found that neuropsychological assessment provided the best prediction of fitness to drive. Clinical interviews were less objective and less standardized than neuropsychological tests and driving simulation. Driving simulation is also not sufficiently predictive if used alone. However, combining all three types of assessments yielded the best prediction for fitness to drive in patients with AD (29). Other systematic review and meta-analysis have demonstrated that executive function, attention, visuospatial function and global cognition revealed by neuropsychological tests may be predictive of driving performance in patients with MCI and AD. Specifically, TMT-A and -B and Maze test emerged as the best single predictors of driving performance though there were variability and inconsistencies. On-road and simulator assessments have yielded inconsistent results in terms of the safety to drive in patients with MCI and AD (3).

From the results of these studies, there has been no single test sufficient to determine driving safety in patients with MCI and AD though the combined use of these tests is somewhat useful. Accordingly, it is necessary to establish an objective method that can be performed easily, in a short time, at a low cost, but has high reliability. Note that ERPs have all such features, therefore, ERPs are suitable for evaluating driving ability in ADS. In the following section, we describe ERP researches on driving evaluation in ADS.

## Assessment of driving ability in ADS using ERPs

ERPs are electrical potential generated by the brain time-locked to a sensory, cognitive, or motor event and provide a powerful, non-invasive technique with superb temporal resolution, for studying the brain's synaptic function (30–32). In general, early ERP components (<200 ms) reflect sensory processes as they depend mainly on the physical parameters of the stimulus, so-called exogenous component. Conversely, later ERP components (>200 ms) are relatively more dependent on the mental operations performed on the stimuli as well as on non-sensory factors such as predictability, higher perceptual and semantic features, so-called endogenous component.

ERPs have been extensively used for functional evaluation of brain in ADS (30–32). The P300 component (around at 300–500 ms) elicited by an oddball paradigm has been most studied in ADS as the convenient measure of the cognitive dysfunction. In general, early sensory components at around 50–100 ms are relatively spared whereas potentials starting around 200 ms and beyond are more consistently abnormal even in the early stage of AD and MCI. Thus, ERPs may reveal neurophysiological changes related to the expansion of the neocortical association areas of AD pathology (32).

For the ERP research on driving, the P300 cognitive component is often used as an index of driving performance in healthy individuals (33–36). However, there have been no P300-ERP studies on driving ability in ADS. To our knowledge, only two ERP studies used N200 component for the driving ability of AD (37, 38) (Table 2). In a study of (37), ERPs were recorded in young and older normal controls, and early AD patients while participants viewed real-world videos and dot motion stimuli (OF) simulating self-movement scenes. In both stimulus conditions, N200 latencies were delayed by aging whereas AD patients exhibited the diminished N200 amplitude. In addition, AD patients were uniquely unresponsive to increments in motion speed. Since OF is crucial for speed judgments and braking during vehicular navigation, the authors proposed that the AD unresponsiveness to accelerations might reveal some of the mechanism involved in their driving impairment and potentially help identify high-risk individuals at earlier stage. In another study (abstract form) (38), early AD patients and older normal control took a virtual reality driving evaluation that incorporates multiple cognitive, visual and motor tests. OF-ERPs were also recorded. Compared to older normal control, AD patients had significantly lower driving scores and smaller N200 amplitudes. Furthermore, there was a highly significant correlation between driving scores and N200 amplitudes. The authors concluded that significant correlations between vehicular driving scores and N200 amplitudes supported the role of extrastriate cortical dysfunction in impaired driving capacity and that the potential use of ERPs as screening tools for selective functional impairments and as biomarkers of AD.

**Table 2:** ERP studies on driving ability in ADS.

**References**	**Participants**	**Study design and protocol**	**Outcome measure**	**Summary of main findings**
Fernandez and Duffy (2012) (37)	- [OF (dot motion)] - Early AD *(n = 15; age, 78.6 ± 8.0)* - Older normal control *(n = 16; age, 76.2 ± 10.0)* - Young normal control *(n = 12; age, unknown)* [Real-world video motion stimuli] - Early AD *(n = 6; age, 73.2 ± 6.3)* - Older normal control *(n = 5; age, 70.6 ± 6.4)* - Young normal control *(n = 9; age, 29.33 ± 8.5)*	- Cross-sectional study - ERPs evoked by OF (dot motion) (Changes of coherence and speed) - ERPs evoked by real-world video motion stimuli (Changes of coherence and speed)	- N200 amplitude and latency	- Diminished N200 amplitude in early AD - Increasing speed elicits smaller N200 amplitudes in early AD
Fernandez-Romero and Cox (2016) (38) (abstract form)	- Early AD *(n = unknown; age, unknown)* - Older normal control *(n = unknown; age, unknown)*	- Cross-sectional study - ERPs evoked by OF - Virtual reality driving evaluation	- N200 amplitude and latency - Multiple cognitive, visual and motor tests	- Smaller N200 amplitude in early AD - Lower driving score in early AD - Significant correlations between vehicular driving scores and N200 amplitudes
Yamasaki et al (1)	- aMCI *(n = 18; age, 72.4 ± 6.9)* - Early AD *(n = 18; age, 75.5 ± 5.7)* - Older normal control *(n = 18; age, 71.8 ± 4.1)* - Young normal control *(n = 18; age, 28.2 ± 5.1)*	- Cross-sectional study - ERPs evoked by OF and HO (dot motion)	- N170 and P200 amplitudes and latencies	- Prolonged latency of OF-specific P200 in aMCI - Prolonged latencies of N170 and P200 in early AD - Significant correlation between OF-specific P200 latency and MMSE score
Yamasaki et al (2)	- aMCI *(n = 15; age, 74.4 ± 4.4)* - Older normal control *(n = 15; age, 73.5 ± 4.5)* - Young normal control *(n = 15; age, 27.9 ± 5.0)*	- Cross-sectional study - ERPs evoked by OF (dot motion), faces, words, chromatic and achromatic gratings	- N170 and P200 amplitudes and latencies for OF - N170 amplitudes and latencies for faces and words - N120 amplitude and latency for chromatic gratings - Steady-state response for achromatic gratings	- Prolonged N170 and P200 latencies for OF in aMCI - Prolonged N170 latencies for faces and words in aMCI - Normal N120 for chromatic gratings in aMCI - Normal steady-state response for achromatic gratings in aMCI - Significant correlations between N170 latency for OF and LM WMS-R scores, and between P200 amplitude for OF and LM WMS-R scores - High AUC in N170 and P200 latencies for OF in ROC analysis

*aMCI, amnestic mild cognitive impairment; AD, Alzheimer's disease; ERPs, event-related potentials; OF, optic flow; MMSE, Mini-Mental State Examination; LM WMS-R, logical memory in Wechsler Memory Scale-Revised; ROC, receiver operating characteristic; AUC, area under the curve*.

These two studies (37, 38) suggest that OF-ERPs (sensory N200 component) may be useful for evaluation of driving ability in AD. However, it remains unknown whether the N200 component is the best predictor of driving ability in AD, and whether or not OF-ERPs can be an index of driving ability even in aMCI. For the last decade, we have been studying the feasibility of sensory ERPs in response to radial OF in aMCI and AD and proposed that OF-ERPs provided an additional information on the alteration of visuospatial perception in ADS (1, 2). The visuospatial deficits (impaired OF perception) related to the posterior temporo-parietal dysfunction play a key role in the navigational or driving impairment in ADS (18, 19, 25). Hence, we hypothesized that sensory ERPs elicited by OF but not P300 cognitive ERPs could be a neural biomarker in driving impairment even in the early stage of ADS. In the following section, we describe neural basis of OF processing in healthy humans and the potential use of OF-ERPs as a driving evaluation method.

## Neural basis of of perception in healthy humans

**Figure 2:**
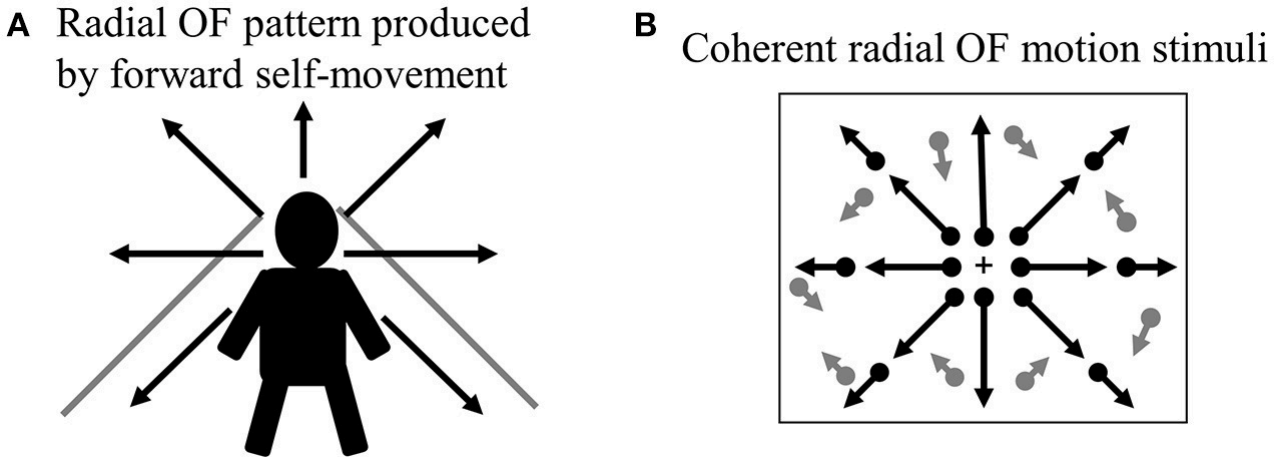
Radial OF motion. **(A)** When we move through our environment, radial OF pattern is produced by forward self-movement. **(B)** Coherent radial OF motion stimuli used in our study. We can create radial OF motion stimuli easily using random dots. Dots radiate from the focus of expansion, which corresponds to the observer's direction of heading. OF, optic flow.

When we move through our environment with walking or cars, the radial pattern of OF is produced at the retina (Figure 2A). The ability of visual motion system that analyzes OF is biologically important because it provides visual cues that can be used to perceive the direction of self-motion, to guide locomotion and to avoid obstacles (20, 39). Thus, the drivers must analyze radial OF information continuously to control his/her vehicle during driving, so that the OF processing is indispensable for safe driving.

**Figure 3:**
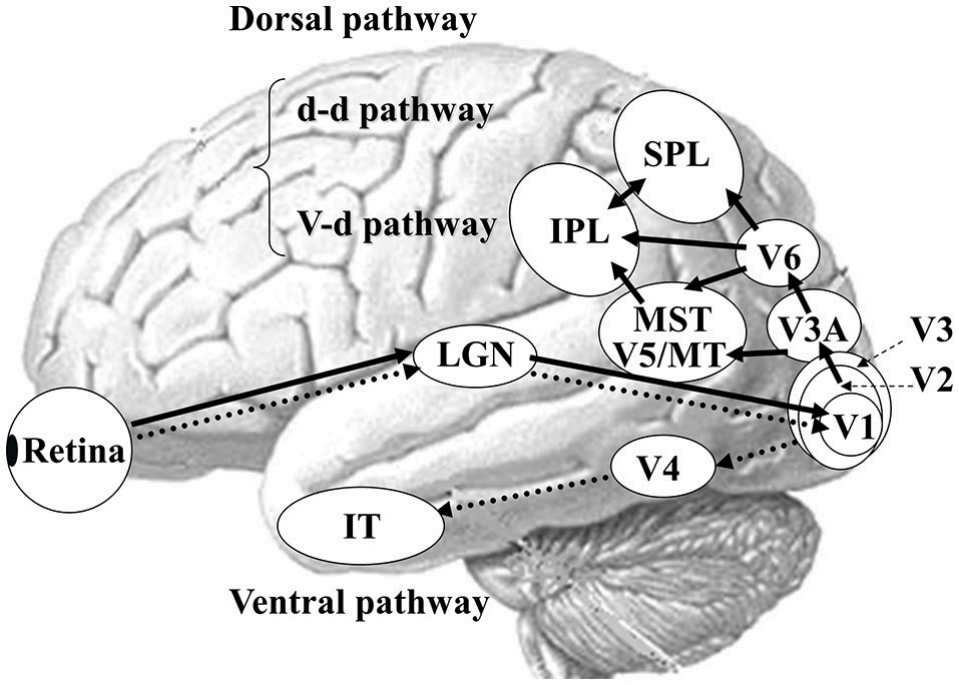
Parallel visual pathways in humans. There are two major parallel streams: ventral and dorsal pathways in humans. Detailed functions of the two streams are provided in the text (see section Neural Basis of OF Perception in Healthy Humans). A recent study has revealed the importance of interconnection between IPL and SPL for OF processing (40) so that we modified this figure considering this point. d-d pathway, dorso-dorsal pathway; v-d pathway, ventro-dorsal pathway; LGN, lateral geniculate nucleus; MT, middle temporal area; MST, medial superior temporal area; IPL, inferior parietal lobule, SPL, superior parietal lobule; IT, inferior temporal cortex. [Modified from (41), Copyright (2012) with permission from IOS press].

In humans, there are two functionally and anatomically segregated visual pathways: the ventral and dorsal pathways (Figure 3) (21, 22, 42). Both pathways begin in the retina and project to the primary visual cortex (V1). After V1, the ventral stream is important for form and color perception, projecting to V4 and the inferior temporal (IT) cortex. In contrast, the dorsal stream is responsible for motion perception, connecting to V5/middle temporal (MT)+ (V5/MT and medial superior temporal area [MST]), V6 and the posterior parietal cortex (21). The dorsal stream also comprises two distinct functional flows; the dorso-dorsal (d-d) and ventro-dorsal (v-d) streams (43). The d-d stream consists of V6 and the superior parietal lobule (SPL) while the v-d stream involves V5/MT and the inferior parietal lobule (IPL). From the concept of such visual processing mechanism, the OF perception is mainly processed by the dorsal stream.

Primate studies have reported a number of cortical areas that selectively respond to OF, including the dorsal part of the MST (44), the ventral intraparietal area (VIP) (45), area 7a (46) as well as area PEc (47). Conversely, V5/MT neurons do not show such specific selectivity (48). In humans, several OF selective areas have been identified by neuroimaging studies within the dorsal streams (49–57). These OF selective areas contain visual areas such as MST and V6, multisensory areas such as the VIP, the precuneus motion area (PcM) and cingulate sulcus visual area, and vestibular areas such as the putative area 2v (p2v) and parieto-insular vestibular cortex (PIVC). A recent fMRI study have demonstrated that the posterior-insular cortex (PIC) area plays an important role in the integration of visual and vestibular stimuli for the perception of self-motion while the PIVC is selectively responsive to vestibular stimulation (58, 59). Overall, the VIP, PcM and p2V are located within the d-d stream (SPL) while the v-d stream (IPL) consists of PIC (40).

## OF-ERPs in healthy humans

**Figure 4:**
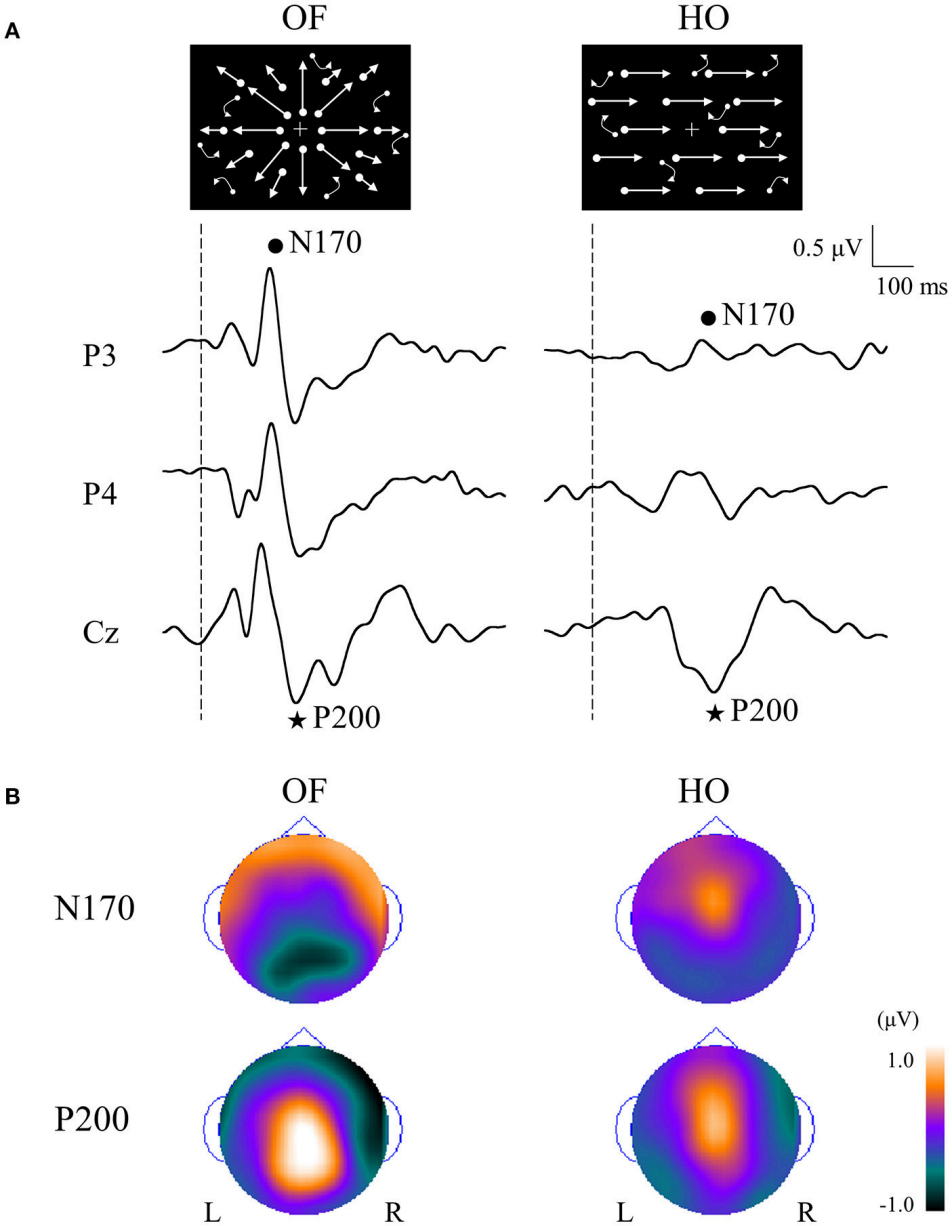
ERPs in response to coherent OF and HO motion stimuli and their scalp topography in healthy subjects. **(A)** It is evident that the N170 and P200 are distinct motion-related components. The N170 component was distributed over occipito-temporal areas regardless of the stimulus type, extending further to the parietal region in the OF condition only. **(B)** The P200 component in response to OF stimuli was distributed over the parieto-central region while that of HO was distributed over the central region. The color bar represents the amplitude value (red = positive, blue = negative). Please note that this figure was presented at 2009 International Symposium on Early Detection and Rehabilitation Technology of Dementia. December 11–12, 2009, Okayama, Japan. ERPs, event-related potentials; HO, horizontal motion.

In order to compare OF processing with HO processing in healthy humans, we recorded ERPs for coherent OF and HO motion stimuli in healthy young subjects by using a high-density EEG system (60) (Figures 2B, 4). We used coherent motion stimuli as the visual stimuli, which consisted of 400 white square dots randomly distributed on a black background. The white dots moved at a velocity of 5.0°/s. Two types of motion stimuli (OF and HO) were used. OF stimuli contained dots that moved in a radial outward pattern while HO contained dots that moved leftward or rightward. The coherent level was 90% in both stimuli. Both stimuli had the same dot density, luminance, contrast and average dot speed. Random motion (RM) was used as a baseline condition. The OF and HO stimuli were presented for 750 ms, with the presentation of RM for 1,500–3,000 ms alternately. The N170 [analogous to N200 in previous ERP studies (37, 38), about 170 ms] and P200 (about 200 ms) were recorded as major components. We analyzed the peak latencies, amplitudes, scalp distribution and the sources in both components.

**Figure 5:**
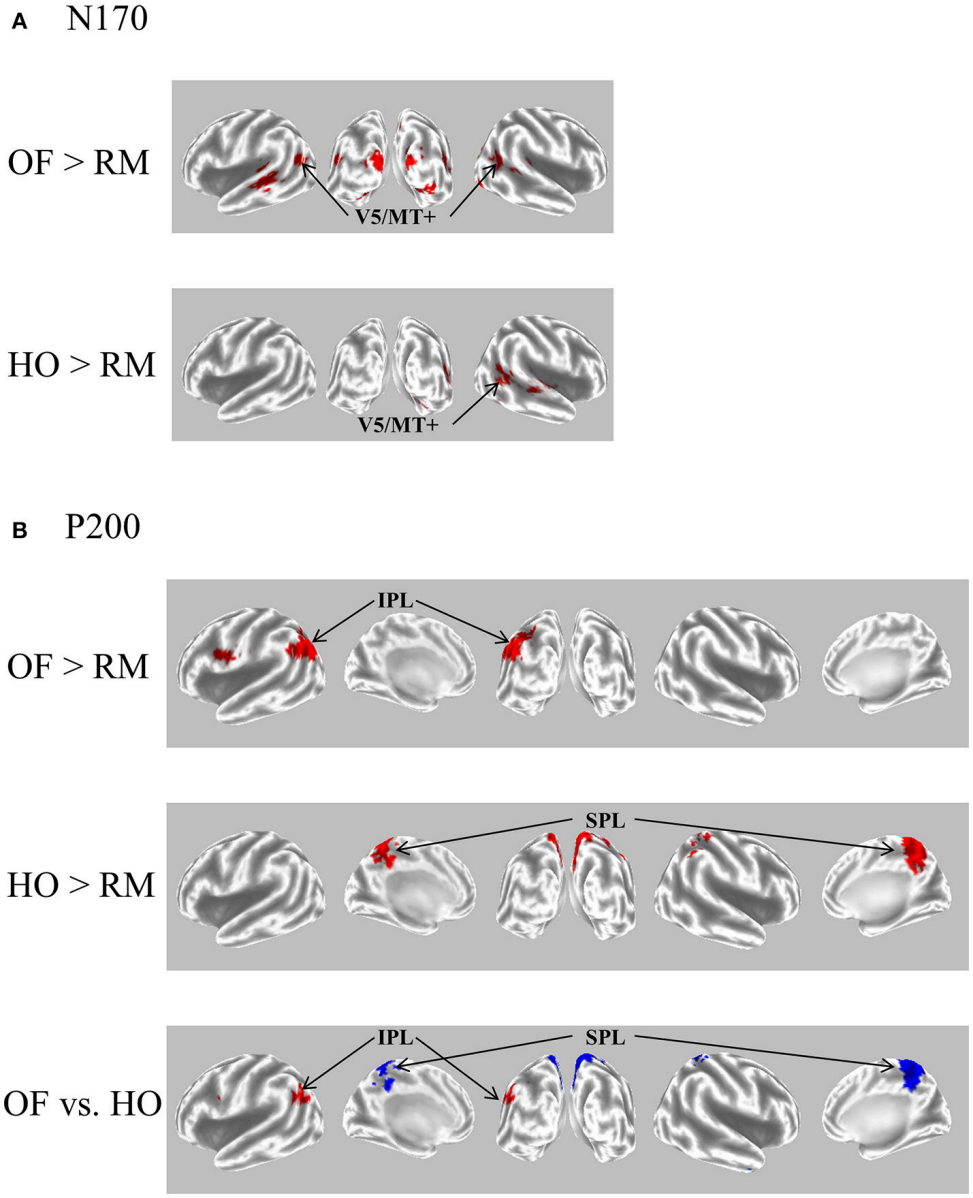
eLORETA-based statistical nonparametric maps for a comparison between OF and HO in GFP peaks of N170 and P200. **(A)** The current density of N170 was significantly elevated over the occipito-temporal areas including V5/MT+ in both stimulus conditions. **(B)** The current density of the parietal P200 for OF was significantly elevated in the left IPL (BA 39/40). Conversely, there was a significant elevation of the current density of the central P200 for HO in the bilateral SPL (BA 7). In the figure at the bottom, red and blue mean OF and HO, respectively. Please note that this figure was presented at 2009 International Symposium on Early Detection and Rehabilitation Technology of Dementia. December 11–12, 2009, Okayama, Japan. eLORETA, exact low resolution brain electromagnetic tomography; GFP, global field power; RM, random motion.

The N170 was distributed over occipito-temporal regions in response to both OF and HO stimuli. The distribution of the OF-N170 extended further into the parietal region compared with those of HO-N170 (Figure 4B). The OF-N170 amplitude was significantly larger and its latency was significantly shorter than those of HO-N170 (Figure 4A). Exact low resolution brain electromagnetic tomography (eLORETA) analysis of the N170 revealed that the current density was significantly elevated over the occipito-temporal areas including V5/MT+ in response to both stimuli compared with RM baseline (Figure 5A). These findings were consistent with those of minimum-norm estimate (MNE) of visual evoked magnetic fields (VEFs) (61). A direct comparison between OF and HO stimuli revealed no significant difference in the current density of the N170. Current density estimation with eLORETA in ERPs and MNE in VEFs provided strong evidence that the generator source of the N170 was located in V5/MT+ for both stimuli. Therefore, the N170 constitutes a non-specific motion component derived from an area close to V5/MT+. However, OF stimuli elicited an N170 with a higher amplitude and shorter latency, compared with HO (Figure 4A), which may reflect a higher activity of V5/MT+ during OF processing. Alternatively, V5/MT+ can be subdivided into V5/MT and MST (50, 62). V5/MT neurons respond to both OF and HO stimuli (48), whereas MST selectively responds to OF (44, 46). Thus, the selective activation of MST neurons may contribute to the higher amplitude and shorter latency of the OF-N170 response.

The P200 component exhibited distinct characteristics between OF and HO. The OF-P200 was distributed over the parieto-central region (Figure 4). HO stimulus also evoked an observable P200, but its topography was limited to the central region (Figure 4). The P200 amplitude was significantly larger for OF compared with HO stimuli. Similarly, the latency of OF-P200 was significantly faster compared with that of HO-P200 (Figure 4A). Regarding the parietal OF-P200, the current density was significantly elevated in the IPL (Figure 5B, top row). In contrast, for the central HO-P200, the current density was distributed over the SPL (Figure 5B, middle row). A direct comparison revealed that the current density of the IPL in response to OF stimuli compared with HO stimuli was significantly elevated (red color). Conversely, the current density of SPL was significantly elevated in HO compared with OF (blue color) (Figure 5B, bottom row). Overall, these findings suggest that the parietal OF-P200 is functionally coupled with the IPL (the v-d stream) and that it is the OF-specific component. Conversely, the central HO-P200 is related to the SPL (the d-d stream) (60). These functional dissociations between IPL (OF perception) and SPL (HO perception) were consistent with our fMRI study (41). Therefore, we propose that different spatio-temporal processing is driven by these motion stimuli within the two distinct dorsal streams in humans. From these findings, it is likely that ERPs with coherent OF and HO motion are useful for functional evaluation of the dorsal stream. More specifically, OF-related ERPs (OF-N170 and OF-P200 components) are considered to be able to identify subtle changes of visuospatial function (OF perception) associated with driving ability in individuals.

## OF-ERPs in ADS

**Figure 6:**
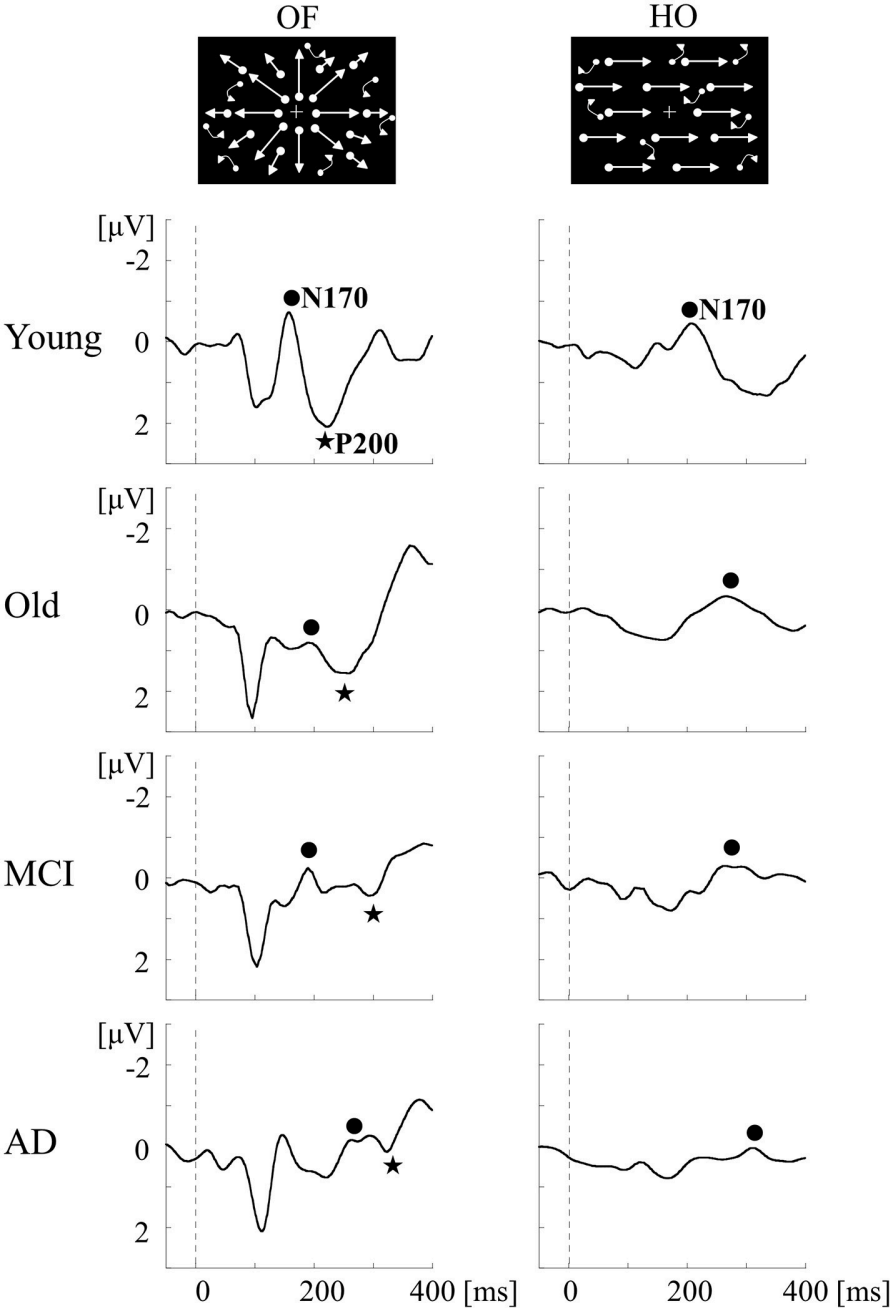
ERPs in response to coherent OF and HO motion stimuli in the MCI, AD and healthy control groups. MCI patients exhibit more prolongation of P200 latency for OF than healthy elderly adults, but no prolongation of N170 latency for both stimuli. AD patients show a prolongation of both N170 and P200 latencies compared with other groups. MCI, mild cognitive impairment. [Modified from (64), Copyright (2012) with permission from IEEE].

To examine whether we can detect the impairment of OF perception in aMCI and AD, ERPs for OF and HO were recorded in patients with aMCI and AD, and in healthy old and young adults (1) (Table 2). aMCI was defined according to the criteria of Petersen (24). The patients with AD met the criteria for probable AD according to NINCDS-ADRDA (63). Neuropsychological tests including MMSE and the Clinical Dementia Rating (CDR) were performed. Regarding ERPs, visual stimuli and analysis were same as the former study in healthy young subjects (60). There was no significant difference in both OF-N170 and HO-N170 responses between aMCI patients and healthy old adults (Figure 6). In contrast, the latency of OF-P200 was significantly prolonged in aMCI patients compared with healthy old adults (Figure 6). Therefore, within the dorsal stream, the v-d stream (IPL) related to OF perception, but not the d-d stream (SPL) associated with HO perception, is selectively impaired in aMCI patients. On the other hand, AD patients showed a prolongation of N170 and P200 latencies for both OF and HO stimuli compared with healthy old adults and aMCI patients (Figure 6). Our results indicate that aMCI patients exhibit a selective impairment of OF perception related to the higher-level of dorsal stream (v-d stream including IPL). Conversely, AD patients show the impairments of both OF and HO perception associated with the distributed higher-level dorsal stream (both v-d and d-d streams including IPL, SPL and V5/MT+). These findings were consistent with the spread of AD pathology following disease progression (1, 64). Thus, we can detect the impairment of OF perception even in patients with aMCI by using OF-ERPs.

**Figure 7:**
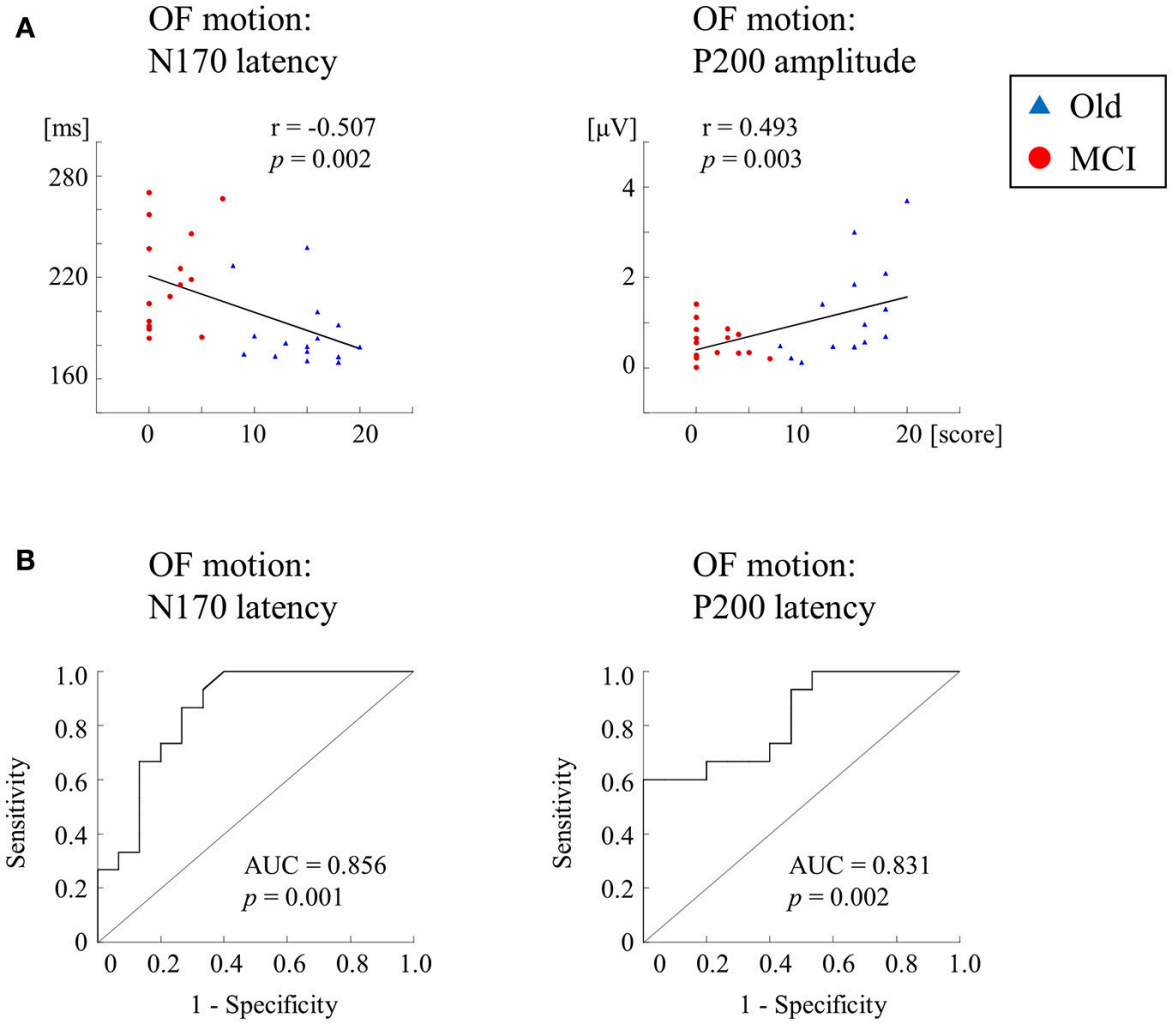
Correlation and ROC analyses. **(A)** Correlation of ERPs with delayed LM WMS-R scores. ERPs for OF motion stimuli are significantly correlated with delayed LM WMS-R scores. **(B)** The results of ROC curve analysis for discriminability of ERP components. The N170 and P200 latencies for OF motion have AUCs ≥ the threshold of 0.7 for acceptable discrimination. Please note that AD group was not recruited in this study [Modified from (1), Copyright (2016) with permission from IOS press]. LM WMS-R, logical memory in Wechsler Memory Scale-Revised; ROC, receiver operating characteristic; AUC, area under the curve.

We further recorded ERPs to multimodal visual stimuli (chromatic and achromatic gratings, faces, kanji and kana words and OF motion) in aMCI patients, healthy old and young adults (2) (Table 2). Inclusion criteria for aMCI patients and healthy old adults followed the criteria of the Japanese Alzheimer's Disease Neuroimaging Initiative (65). These criteria were based on several neuropsychological tests: MMSE, CDR, Geriatric Depression Scale and the logical memory test (delayed recall) of the Wechsler Memory Scale-Revised (WMS-R). Multimodal visual stimuli were optimized to activate elements of each visual stream separately. The OF stimulus was same as the former studies (1, 60). ERP responses to lower (V1) level stimuli (chromatic and achromatic gratings) were not significantly differed between aMCI patients and healthy old adults. Conversely, ERP latencies for higher-ventral (faces and kanji words) and higher-dorsal (kana words and OF motion) were significantly prolonged in aMCI patients. Interestingly, OF-related ERPs were significantly correlated with the logical memory test (delayed recall) of the WMS-R (OF-N170 latency, *r* = −0.507; OF-P200 amplitude, *r* = 0.493) (Figure 7A). Furthermore, the receiver operating characteristic (ROC) analysis exhibited that the highest area under the curve (AUC) was observed for OF-ERP latencies (OF-N170 latency, AUC = 0.856; OF-P200 latency, AUC = 0.831) (Figure 7B). This suggests that OF-ERPs have the best distinguishing ability between aMCI and healthy old adults.

## A potential use of optic flow-ERPs in assessing driving ability in ADS

Overall, in our ERP studies (1, 2), OF-related visuospatial perception indispensable for driving was associated with cognitive function in ADS. As previously mentioned, severity of decline in driving ability was correlated with the degree of cognitive function (23) or visuospatial function (17). Therefore, we assume that OF-ERPs can detect early signs of decline in driving ability in patients with ADS.

In support of our view that altered OF-related visuospatial perception is associated with the driving disability in ADS, Vilhelmsen et al. (66) found that the latency of N2 (analogous to N170 in our study) increased as the speed of OF-motion increased (driving speeds 25, 50, and 75 km/h) in healthy young subjects. They supposed that the subjects perceived the OF stimulus with higher speeds as more complex than that of the lower speeds, which resulted in the increased N2 latency. Healthy individuals can handle our OF stimulus easily but the damaged ADS brain may need more effort because of an excessive load for the visuospatial processing system. This interpretation may explain the delayed N170 and P200 latencies in our study (1, 2).

Based on ERP findings of our (1, 2) and other groups (37, 38), OF-ERPs (both N170 and P200 components) may be useful for evaluation of driving ability in aMCI and AD patients. However, it should be kept in mind that the relationship between OF-ERPs and the performance of on-road and driving simulator tests has not established. In addition, we have not yet determined the reference values of OF-ERPs (amplitude and latency) on driving ability. Thus, in the near future, we will perform a large-scale longitudinal ERP study for determining the relationship between driving ability and OF perception in a wide range of ADS. By doing so, we can assess driver's aptitude to prevent the traffic accidents in patients with ADS. Meanwhile, we are currently trying to develop the simple and reliable touch panel-type assessment system of driving ability using radial OF stimuli (measuring OF-detection threshold) (https://kaken.nii.ac.jp/en/grant/KAKENHI-PROJECT-17K09801/). This system may be useful for driving performance evaluation, which is much simpler than ERPs.

## Conclusions

To maintain safe driving, widespread brain networks including occipital, parietal, frontal, motor and cerebellar regions are recruited. These brain networks are vulnerable in ADS pathology that shows extensive neocortical brain damage. In ADS, the driving ability continues to gradually decline accompanied by the course of AD pathology. Especially, the early pathological change in the posterior temporo-parietal regions related to OF perception is responsible for the impaired driving in the early stage of ADS. Although various methods including on-road test, driving simulation and neuropsychological tests are used for evaluating driving ability, there is no single test sufficient to determine driving safety in ADS patients. Conversely, ERPs are non-invasive and objective method that can be performed easily, in a short time, at a low cost, but has high reliability. Based on previous and our ERP studies, OF-ERPs can be an indicative neural biomarker for assessing the decline of driving ability in ADS.

## Author contributions

All authors listed have made a substantial, direct and intellectual contribution to the work, and approved it for publication.

### Conflict of interest statement

The authors declare that the research was conducted in the absence of any commercial or financial relationships that could be construed as a potential conflict of interest. The reviewer DS and handling editor declared their shared affiliation.
